# Cocaine induced acute lung injury: A Case Report

**DOI:** 10.51894/001c.144209

**Published:** 2025-09-30

**Authors:** Arfa Ali

**Affiliations:** 1 Internal Medicine Detroit Medical Center-Sinai Grace Hospital

## 60

### INTRODUCTION

Crack lung or cocaine-induced lung injury is an acute pulmonary syndrome characterized by diffuse alveolar damage occurring within 48 hours of smoking crack cocaine. Clinical and radiologic features vary, making it crucial to have high suspicion of such cases to direct management. Herein is a case of a 61-year-old female presenting with shortness of breath.

### CASE PRESENTATION

61-year-old female with history of hypertension, diabetes mellitus and polysubstance use disorder presented with shortness of breath. Patient admitted symptoms developed about 12 hours after using crack cocaine. She had no prior history of chronic lung disease, although she smoked a half-pack per day for 41 years. Presenting vitals were: blood pressure 166/96, heart rate 116, respiratory rate 28, temperature 37, and pulse oximetry 77% on room air, improving to 100% on 15 L via non-rebreather mask.

Exam revealed patient in respiratory distress and speaking in 1–2-word sentences; auscultation revealed bilateral wheeze, with remainder of the exam being unremarkable.

Significant labs were: leukocytosis (WBC 21.4) with neutrophilia and presence of cocaine, methadone and opiates on urine toxicology. Chest x-ray and CT chest revealed pulmonary edema and extensive airspace disease respectively. Predominant lung findings were those of “crazy paving”, which is diffuse areas of ground-glass opacity surrounded by septal thickening.

Patient was placed on high flow nasal cannula and started on systemic steroids as well as ceftriaxone and azithromycin empirically. She was transitioned to oxygen via nasal cannula following rapid improvement. By day 4 of admission, she was saturating 94% on room air. She was discharged with a prednisone taper and asked to follow up with pulmonology for likely underlying COPD.

### DISCUSSION

Crack lung commonly presents soon after cocaine inhalation and patients may present with acute onset dyspnea, tachypnea, wheeze, cough, hemoptysis or hypoxemic and/or hypercapnic respiratory failure. Combination of detailed history and pertinent investigation findings from CBC, UDS, CXR and CT chest can help make a timely diagnosis.

### CONCLUSION

Crack lung can rapidly progress to ARDS if timely identification and intervention are not initiated. Presentations vary but supportive therapy with supplemental oxygen and anti-inflammatory agents can result in rapid improvement of symptoms.

